# Analyzing systemic lupus erythematosus publications using neural network–based multi-label classification algorithms

**DOI:** 10.1177/09612033221093548

**Published:** 2022-04-13

**Authors:** Enayat Rajabi, Maryam Sahebari, Tressy Thomas

**Affiliations:** 1 Shannon School of Business, 55964 Cape Breton University, Sydney, NS, Canada; 2 Rheumatic Diseases Research Center, Mashhad University of Medical Sciences, Mashhad, Iran; 3 55964 Cape Breton University, Sydney, NS, Canada

**Keywords:** Lupus, SLE, Systemic lupus erythematosus, multi-label text classification, deep neural networks, convolutional neural networks

## Abstract

The heterogeneity in systemic lupus erythematosus research topics poses a challenge for the entire lupus community, from basic geneticists to clinical investigators. As such, it is critical for medical professionals to remain up to date on directions in lupus research and the main fields in which this research is being conducted (e.g., etiology, diagnosis, treatment, and outcomes). This article develops two multi-label text-classification models using Deep Neural Networks and Convolutional Neural Networks to classify the human-based adult-onset lupus–related articles in the PubMed database based on their abstract, keywords, and MeSH terms. During training evaluation, our models correctly indicated all relevant labels for 70% of the articles. The applied machine learning models (Deep Neural Network and Convolutional Neural Network) yielded a Micro-F1 score of 0.89, meaning that it successfully labeled the most relevant medical domains and types. In addition, these types of studies help the researchers be aware of the essential topics in this field, but due to difficulties in designing, the related studies are ignored or fade.

## Introduction

Systemic lupus erythematosus (SLE) or “lupus” is an autoimmune disease with unknown etiology and several morbidities. The inflammation caused by lupus can affect many body systems, including joints, skin, kidneys, blood cells, brain, heart, and lungs. The heterogeneity of SLE poses an ongoing challenge for the entire lupus community, from geneticists to clinical investigators, as it extends beyond clinical assessment into laboratory findings, which raises complex issues concerning the classification, diagnosis, and treatment of the disease. Moreover, the etiology and pathogenesis of SLE have become a hot topic in the medical sciences.^1,2^ In addition, treatment strategies for lupus are largely experimental, and there is no standard therapy for several manifestations of the disease.^3^ As such, medical professionals need to remain up to date on the latest directions in lupus research, especially regarding the main areas of study, including etiology, diagnostic criteria, treatment, and outcomes. It is also essential to consider the type of article when conducting a scientometric analysis of a topic, as systematic reviews and meta-analyses are more significant than expert opinions and case reports in such studies. One of the main methods of identifying the most recent areas of lupus research is to search for articles published in this domain using different databases available on the Web. For example, the PubMed database contains thousands of lupus-related entries,^4^ each containing information such as the article’s title, an abstract, publication type, publication year, and so on. Beyond that, PubMed categorizes articles using keywords and the Medical Subject Headings (MeSH),^5^ a comprehensive controlled vocabulary developed and maintained by the National Library of Medicine to index the various topics in each abstract PubMed database. However, many published articles have not been labeled with corresponding MeSH terms. Moreover, some articles were assigned numerous MeSH terms from different categories, making precise classification nearly impossible. As a result, a medical professional seeking to research lupus would need to review the abstract of each article to determine which categories it properly belongs to, for example, for etiology, treatment, or diagnosis.

This article addresses the lack of a system for classifying lupus articles in the PubMed database by developing a multi-label classification technique that will enable lupus professionals and researchers to identify current directions in lupus research easily and quickly.

Specifically, in this paper, we make the following contributions:

1. We write a software tool capable of collecting a set of lupus articles based on defined search keywords and a set of criteria.
2. The collected articles are then cleaned, structured, and labeled by a domain expert.
3. We analyzed all the labeled articles based on a defined category, type, number of authors, and their affiliation.
4. Finally, we develop two neural network–based machine learning algorithms capable of predicting the domain area and type of lupus articles.

The structure of this article is as follows: Background section provides an overview of this research and related works; Materials and Methods section discusses the data-collection process, materials, and advanced method; Results and Discussion section outlines the results and the discussion in further detail, and Conclusion and Future Works section provides some general concluding remarks.

## Background

Expanding the types and number of lupus therapies and ongoing basic or translational research can improve individualized medical care and outcomes. The use of advanced analytics techniques to identify in-demand areas of lupus research can provide new insights into the science of the disease.^6–8^ This is critical, as medical professionals and researchers of autoimmune diseases with unknown etiologies need to remain up to date on the direction of research in their given fields. Tools that enable the classification of published lupus articles allow medical researchers to identify when and why a specific topic began to attract attention, the most active sub-topics, and the developments that have taken place up to the present. Moreover, the ability to categorize lupus articles (type-wise or class-wise) can help medical researchers identify which areas of lupus research are most popular and which require more attention. Although leveraging machine learning algorithms models has become quite common in the medical domain, we are unaware of any studies that focus on classifying lupus research publications based on their titles and abstracts.

Developing a classification system would provide researchers with a tool that can help them obtain their desired search results effectively. In this research, we design a classification system that assigns a medical category to each article (with a probability of belonging the article to that category). This system can subsequently be incorporated into the PubMed database’s (along with the other public databases on the Web) search mechanism to allow users to filter search results based on an article’s medical categories. Likewise, these databases can also incorporate article type into their search mechanisms to provide more efficient search results.

Multi-label text classification models analyze a textual comment and predict multiple labels associated with the comments. Given that more than one class or label (etiology, diagnosis, etc.) is assigned to an article in this study, this falls into a multi-label text classification problem. A machine learning model should predict different categories or labels of an article based on its title and abstract. We perform a literature review to identify the emerging machine learning models for a multi-label text classification problem. We used the PubMed database to search for recent studies that leveraged multi-label classification models in the medical context. We reviewed 20 articles and selected the most related ones to consider for developing two machine learning models in our study. Table 1 shows five approaches that used multi-label text classification models in the medical or clinical domain, along with the research objective and the approach used. According to our investigation, different types of neural network–based models (i.e., Deep learning and Convolutional Neural Network) were the prevalent and efficient machine learning models used for text classification problems. It should be noted that the developed multi-label text classification model in this study can also be used in any research publication database to improve their search results.

**Table 1.** Recent related studies in medical and clinical text classification.

Study	Model	Method
Medical text classification using convolutional neural networks^9^	Sentence-level classification of medical texts	CNN
Clinical text classification with rule-based features and knowledge-guided convolutional neural networks^10^	Disease classification	CNN
Applying deep neural networks to unstructured text notes in electronic medical records for phenotyping youth depression^11^	Youth depression phenotyping	Deep learning
FasTag: Automatic text classification of unstructured medical narratives^12^	Cohort identification	LSTM-RNNs
Binary classification of Lupus scientific articles applying deep ensemble model on text data^13^	Lupus scientific articles classification	Ensemble deep learning models

## Materials and methods

**Figure 1.**
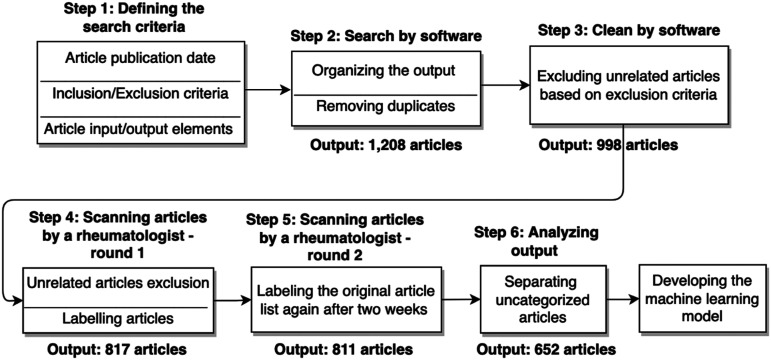
Search methodology.

In an attempt to evaluate the status of knowledge and directions of research concerning lupus, we investigate recent lupus publications (published in 2019 and 2020) in the PubMed database using an emerging machine learning algorithm. Figure 1 shows our search methodology to collect and prepare research articles for developing the model. After extracting the main attributes of each article, we retrieved the main elements of articles including title, abstract, keywords, authors’ names, and their affiliations. Our search methodology consists of the following steps:

1. Collecting lupus articles based on a set of criteria and keywords using a software and PubMed API^1^ (results in 1208 articles)
2. Cleaning the dataset by removing unrelated articles by searching in their titles, abstracts, MeSH terms, and keywords (results in 998 articles)
3. Removing duplicates and reviewing the articles manually by a domain expert (results in 811 articles)
4. Labeling the articles based on their categories and their type by a domain expert (results in 652 articles)
5. Conducting exploratory analysis on the dataset concerning medical category/domain, type of publication, affiliated organization, authors and Normalized Citation Impact Index (NCII)
6. Developing a machine learning model (a multi-label text-classification algorithm) to classify the articles based on their title, abstract, keywords, and MeSH terms
7. Predicting recently published articles based on the implemented algorithm

Different statistical methods are commonly applied to investigate various quantitative or qualitative aspects of a publication in terms of machine learning methods. In medicine, large amounts of scientific information that is difficult to classify are produced every day. Recently, medical researchers and professionals in various fields have increasingly used neural network–based machine learning algorithms to gain insights into specific diseases. In the following subsections, we will explain how to prepare data for modeling and develop the machine learning model.

### Data collection

As mentioned earlier, we defined a set of search keywords and inclusion/exclusion criteria to identify all lupus-based articles. The inclusion criteria are as follows:

1. The articles should be primarily written in English.
2. Articles should include “Systemic lupus erythematosus OR Lupus OR SLE” in their title, abstract, and keywords.
3. Articles should have been published between 2019–01 and 2020–12.

The PubMed database was accessed using the Entrez API^2^, an integrated, text-based search and retrieval system used at the National Center for Biotechnology Information for major databases such as PubMed, Nucleotide, Protein, Genome, and Taxonomy. We used Python programming language to retrieve the main elements of articles from the PubMed database, including the article’s title, abstract, keywords, MeSH terms, publication year, and authors’ information such as authors’ names and their affiliations.

Given that the focus of this study is investigating human-based adult onset lupus-related articles, we excluded the following keywords from the search: “juvenile,” “adolescent,” “rat,” “wolf,” “mice,” “mouse,” “animal,” “child,” “children,” “baby,” “pediatric,” “neonatal,” “newborn,” “girl,” “boy,” “toddler,” and “canis.” The clean dataset was later structured for the exploratory analysis. At the end of this phase, 652 articles were used for labeling and manual review.

### Data labeling and analysis

The collected publications were manually classified according to their types (original article, meta-analysis or systematic review, case report letter or short common, and review article) and their medical category (etiology, diagnosis, symptoms, treatment, and outcome) by a rheumatologist (the second author of this paper). This domain expert assigned each article to one or more categories based on its title, abstract, keywords, and existing MeSH terms. It should be highlighted that the domain expert (a rheumatologist and the second author of this paper) reviewed the list of articles by their title and abstract in two separate rounds at different times to ensure that the review results are accurate. In this manual review, several articles were identified unrelated to the subject of this study and excluded. The exclusion reasons were finding articles related to animals or childhood or different domains (SLE in the military) that recognized by the software as “related articles” incorrectly. The analysis of articles also revealed that a particular test of pure antiphospholipid disease, called “Lupus anticoagulant,” was considered a related article by the software. We excluded those articles from our articles manually.

**Figure 2.**
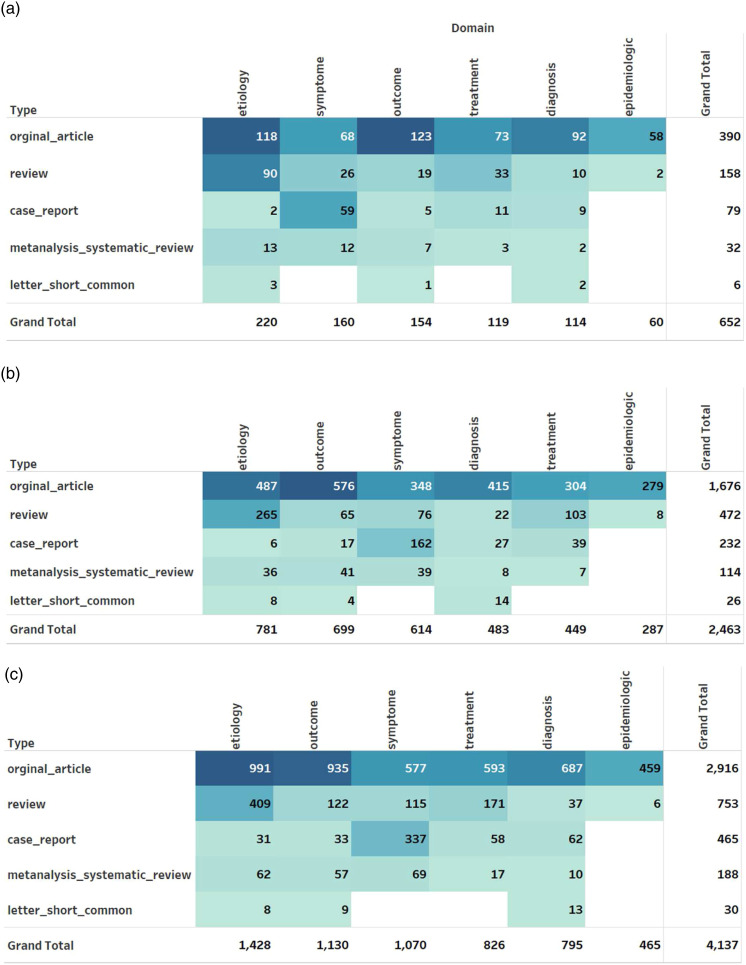
(a) Distribution of articles, (b) affiliations, and (c) authors by domain and type of article.

After labeling and cleaning the final dataset, we performed a quantitative analysis to analyze the number of articles published in each category. As can be seen in Figure 2(a), most of the reviewed publications were original articles (60%), followed by review articles (24%) and case reports (12%). Most original articles were in the outcome, etiology, and diagnosis categories respectively. Most case reports focused on symptoms, while most reviews focused on etiology followed by treatments. Figure 2(b) also presents the number of affiliated organizations associated with each type and category of publication. There is a total of 2463 organizations. From organizations’ contribution perspective, most publications were on the etiology (32%) and outcome (28%) categories. While the review type articles are in the etiology (56%) and treatment (21%) domains, case reports focused on symptoms (70%) followed by diagnosis (12%). In terms of systematic reviews and meta-analysis, most organizations worked on symptoms, outcomes, and etiology domains. The below institutions that published three lupus-related studies during the aforementioned period was the top organization that contributed to lupus research

1. Department of Immunology and Rheumatology, Instituto Nacional De Ciencias Médicas Y Nutrición Salvador Zubirán, Mexico City, Mexico.'
2. Rheumatology Research Group, Institute of Inflammation And Ageing, University of Birmingham, Birmingham, UK
3. Medical School, Universidad Icesi, Cali, Colombia
4. Division of Rheumatology, Department of Internal Medicine, Yonsei University College of Medicine, - Yonsei-ro, Seodaemun-gu, Seoul, Republic Of Korea

From the department of researchers’ perspective, most lupus studies were undertaken by the department of Rheumatology, followed by medicine/internal medicine. Other significant contributors were Dermatology, Pathology and Nephrology.

Figure 2(c) shows the number of authors associated with domain and type of article. There are 4137 authors associated with the 652 articles we have studied. The top authors during 2019 and 2020 in the lupus research from the dataset are identified as Hai-Feng Pan and Andrea Doria. Hai-Feng Pan published eight papers which were in the etiology area. Andrea Doria published seven articles and they were mainly in diagnosis and outcome. To evaluate the impact of articles based on the publication’s longevity, we have used the metric NCII.^14^ The NCII considers the longevity of a publication which refers to the number of years the publication has been in print.

$$NCII=\frac{Number\;of\;citation\;per\;publication}{\left(Number\;of\;years\;since\;publication\right)\;}\;$$

**Figure 3.**
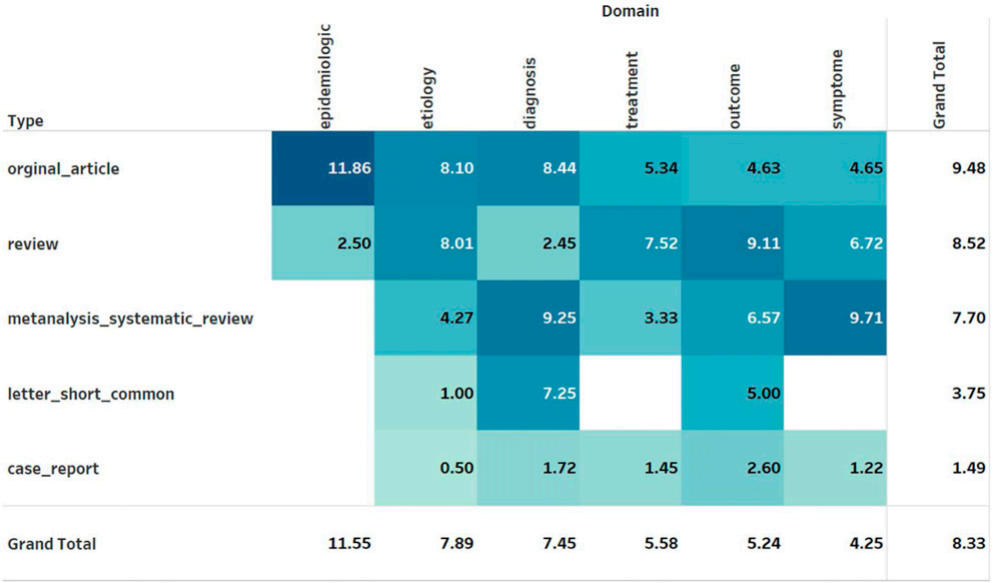
(a) Average Normalized Citation Impact Index Score.

Figure 3 shows the average NCII score for the domain and type of articles. In general, the articles published in the epidemiologic domain recorded higher NCII scores. Articles of type original and review had relatively high NCII score and case report the least.

The article “European league against rheumatism American college of rheumatology classification criteria for systemic lupus erythematosus” received the highest NCII of 406.5.

### Preparing the dataset

We cleaned the text of the articles (i.e., title, abstract, keywords, MeSH terms, the authors’ information, and their affiliations) using the following approach for data analysis and modeling:

1. Merging title, abstract, keywords, and mesh terms as a single text
2. Removing all records where any row contains a null or empty value
3. Removing all special characters and stop words
4. Cleaning and harmonizing the target labels
5. Converting the text into lower case
6. Lemmatization of the word to the root word

Finally, we transformed each article’s text into a sequence of numbers to be fed into the models. The sequences of the numbers were padded to the same length (200).

### Data model

Given the existence of multiple mutually non-exclusive outputs (multiple categories), we followed a multi-label classification approach to predict an article’s domain and type based on its title, abstract, keywords, and mesh terms. To this end, we developed two machine learning models using neural networks. We used Deep Neural Network (DNN), which is a feed-forward neural networks with hidden layers wherein data move only from input to output. We also developed a Convolutional Neural Network (CNN) which is a category of deep learning methods, wherein convolution layers are used for filtering and adaptive learning of the input features by utilizing fully connected layers with back propagation. Both models were trained using a training set size of 436 and test set size of 216. The

The code for these models is available on Github^3^.

### Evaluation metrics

The model’s performance was evaluated based on the accuracy of the metrics, precision (weighted), recall (weighted), F1 Score (micro and macro), and Hamming Loss on the training and testing data. Since this is a multi-label classification problem, the notion of partially correct predictions must also be considered. To this end, we employ the Micro and Macro F1 scores and Hamming Loss. Furthermore, the results were also evaluated by a domain expert to verify the model’s performance.

Accuracy was measured as the ratio of the total number of samples with exact predictions to the total sample size.

Precision $P_{i}$ , Recall $R_{i}$ , and F1 Score $F_{i}\;$ , for a class $C_{i}$ is defined as

$$P_{i}\;=\;\frac{t_{pC_{i}}}{t_{pC_{i}}+f_{pC_{i}}}$$

$$R_{i}\;=\;\frac{t_{pC_{i}}}{t_{pC_{i}}+f_{nC_{i}}}$$

$$F_{i}\;=\;\frac{2.P_{i}R_{i}}{P_{i}+R_{i}}$$

$$Micro\;F_{i}\;=\;\frac{2.MicroP_{i}MicroR_{i}}{MicroP_{i}+MicroR_{i}}$$

$$Macro\;F_{i}\;=\frac{1}{L}\sum_{i=1}^{L}F_{i}\;\;$$

where $t_{pC_{i}}$ is the number of true positives, $f_{pC_{i}}$ is the number of false positives, and $f_{nC_{i}}$ is the number of false negatives. Hamming Loss computes the symmetric difference between the predicted labels and the relevant labels and calculates the fraction of this difference in the label space.

## Results and discussion

The performance of the models is provided in Table 2. As is evident from the metrics, both models had approximately identical accuracy scores, while the DNN model outperformed the CNN model concerning all other metrics. The precision score for both models was more significant than 0.7, which indicates a low false-positive rate. The DNN model had a better recall score than the CNN model, which means that it performed better with respect to correctly identifying labels. Finally, the DNN model’s better F1 scores and Hamming Loss confirm its superiority to the CNN model. We have used the titles, abstracts, keywords, and mesh terms of articles as input to the model. The addition of other input fields such as affiliation did not result in better performance. The tool predicted article type, with results showing match rates of 93% for case reports, 96% for the letter short common, 58% for original articles, and 79% for the review articles. Our model’s predictions matched those of the reference tool 85% of the time.

**Table 2.** Evaluation metrics of the models.

Model	Accuracy	Precision	Recall	Macro-F1	Micro-F1	Hamming loss
DNN model	0.7408	0.9378	0.8646	0.8694	0.8993	0.0403
CNN model	0.7393	0.9589	0.8472	0.8835	0.8990	0.0396

The developed tool in this study could be used to discern which fields of study researchers in the lupus context were most interested in based on articles published in scientific databases (e.g., PubMed) to identify under-researched areas. In addition, this tool can be used to identify articles that have been missed due to their scores and importance or due to the heterogeneous structure of the studies.

The autoimmune diseases related to rheumatology are challenging to diagnose and treat. They have special and complex features. Nonetheless, the most important characteristics of this group of diseases are followed:

• They usually present as multi-system diseases
• Their etiology is usually unknown
• They caused by a combination of various environmental, genetic, infectious, or other risk factors
• They have various manifestations in different populations

Therefore, there is no uniform approach to treat these diseases, and in some cases, there are no known treatments.

As a result, some international organizations have attempted to provide guidelines based on the aggregation of global research, the classification of information obtained, and experts' opinions, which are aimed at achieving the best results concerning diagnosis and treatment for these diseases. Examples of these organizations include EULAR (European League Against Rheumatism), ACR (American College of Rheumatology), and APLAR (Asian and Pacific Area League Against Rheumatism).

In general, the world’s science academies usually determine the methods and direction of study for a disease and scoring articles for preferred publication. For example, the tendency to publish case reports and case series has decreased due to their relatively low citations and the high importance assigned to clinical studies by academic journals because of higher impact of academic scores and citetions. This shift may not harm the scope of information or remove ambiguities about several diseases with long experiences and well-known manifestations, such as gastric ulcer disease or fractures and trauma. However, the Covid pandemic has shown how critical it is to share information about the clinical and paraclinical manifestations of emerging and unknown diseases as case reports and case series, as doing so can be instrumental in developing a better understanding of their etiology and associated treatment methods, which is the main goal of this research domain.

In examining the statistics from our review of lupus-related articles, it became apparent how much the number of case reports has decreased, despite the emergence of several new manifestations and ongoing debates about the clinical, laboratory, and treatment strategies for this disease. Indeed, the policies of journals are very effective in preventing these types of articles from being accepted. Our findings also revealed that most case reports focus on lupus symptoms; conversely, few case reports discuss the results of treatments, which represents a significant gap in the literature. Furthermore, it is complicated to conduct open-label, cross-over studies and RCT (Randomized Clinical Trial) studies to evaluate drug and treatment effectiveness in cases in which a disease is treated using a combination of therapeutic approaches. Hence, conducting systematic reviews or metanalyses of the literature related to such diseases is also challenging.

Thus, policymakers should make annual proposals, informed by international registries, to improve the treatment research process and yield more generalizable results concerning the treatment of lupus various manifestations. The better inclusion and exclusion criteria are defined for all researchers by policymakers and journal systems, better RCTs and other treatment-based research are run, and better systematic reviews and metanalyses will be carried out in the future.

Moreover, we encourage the journal and conference systems to leverage machine learning tools, similar to the developed software in this study, to categorize the types and categories of submitted articles. This will allow researchers to search for related articles effectively. For example, PubMed indexes most original articles, systematic reviews, and letter articles as “articles,” without categorizing human or animal studies which means that a researcher would need to check the title or abstract of papers to determine whether it deals with the type of the article and human or animal studies. The proposed tool in this study can be also used in the same way to add the other categories such as “animal study” to the keywords list of lupus articles.

## Conclusion and future works

In this study, we attempted to identify the main contemporary fields of human-based adult-onset lupus research, including etiology, diagnosis, treatment, and outcomes, to enhance medical professionals’ and researchers’ access to this information. To this end, we developed a tool to collect lupus-related articles from the PubMed database classify them based on their abstract, keywords, and MeSH terms using two multi-label text classification models (DNN and CNN). Our models could correctly indicate all relevant labels for 70% of the articles. The performance of the models can be improved by training them with more records. Notably, most identified recent publications were original and review articles focused on the etiology and outcome of lupus. The following conclusions were derived from this study:

1. This study shows that improving publication databases help researchers to perform more efficient searches and facilitate their access to the related publications. For example, identifying the articles related to animals or specifying the type of articles when authors submit their papers (e.g., original articles, reviews, and case reports) are some of these implications.
2. Analyzing the number of articles published in the scope of this study indicates that there are only a few systematic reviews and meta-analysis articles published during the last years in each category. Enforcing common and essential policies and strategies to make the original researches or treatment studies eligible for entry in meta-analysis facilitates writing more systematic reviews or meta-analysis articles.

## Notes

1. https://pypi.org/project/pymed/ Accessed December 16^th^, 2021

2. https://www.ncbi.nlm.nih.gov/home/develop/api/ (accessed 8, August 2021)

3.
https://github.com/erajabi/Multi_Label_Classification_Lupus
